# Access to medicines for acute illness and antibiotic use in residents: A medicines household survey in Sichuan Province, western China

**DOI:** 10.1371/journal.pone.0201349

**Published:** 2018-08-16

**Authors:** Pengqing Deng, Jianlong Yu, Naitong Zhou, Ming Hu

**Affiliations:** West China School of Pharmacy Sichuan University, Chengdu, Sichuan, China; Peking Union Medical College School of Public Health, CHINA

## Abstract

**Objective:**

To investigate medicine accessibility and antibiotic use in households in western China using World Health Organization (WHO) methodology as well as to identify the influencing factor of care-seeking outside the home and primary determinant factors that influence the use of antibiotics in Chinese residents.

**Methods:**

A cross-sectional household survey was conducted from March to July 2015, and 1200 households from six cities in Sichuan China were selected for a questionnaire survey using stratified multistage random cluster sampling. We used logistic regression models to identify the determinants of care-seeking outside the home and subsequent antibiotic use among the surveyed residents.

**Results:**

Overall, 1103 valid questionnaires were collected, and 458 households reported that they had had experienced at least one acute illness in the previous 2 weeks. Of these households, 97.2% of individuals with acute conditions sought care outside their homes and 40.1% of individuals who took medicine received antibiotics. Only 15.9% of the individuals with acute conditions reported that the medical insurance reimbursement covered at least one medicine. According to the multivariate analyses, women were less likely to seek care outside the home compared to men. Among those who sought outside care, fever and upper respiratory symptoms increased the odds of taking antibiotics, and visiting a private hospital also increased antibiotic use. Low-income households were less likely to receive antibiotics. Symptoms were strong determinants of antibiotic use when patients sought outside care.

**Conclusion:**

The accessibility of medicine for acute illness among households in western China was favorable; however, medical insurance reimbursement must be improved. The nature of the symptoms and patterns of care-seeking had the greatest influence on the decision to take antibiotics among residents with acute conditions. The percentage of antibiotic use in patients with acute illness has declined, but the indications for using antibiotics must be standardized.

## Introduction

Acute health conditions are characterized by the sudden onset of and rapid change in symptoms, a typical course of illness, and a finite duration if the illness is self-limiting or effectively treated.[1] Typically, people are more likely to self-treat acute health conditions than seek medical care, particularly those that occur frequently. However, inappropriate self-treatment may bring some negative consequences, such as masking symptoms, delaying professional health care, having an incorrect diagnosis, or being exposed to unnecessary medications.[2] It is important for patients with acute conditions to have access to affordable and safe medicines and the ability to rationally use these medicines.

Equitable access to essential medicines is considered to be a fundamental human right by the World Health Organization (WHO) and many national constitutions.[3] For many in China, poor access to medical services and high cost for medicines were problems that residents most concerned about, and even today, these issues trigger the most intense response.[4, 5] Since 2009, the Chinese government has implemented a series of health reform policies to improve the accessibility of medicine; one important policy was the National Essential Medicines System (NEMS).[6] According to the Chinese government, to increase the accessibility to essential medicines, such medicines were centralized online, subject to open bids and purchased at the province level, with unified distribution to health facilities. Community health service facilities and grass-roots health care facilities managed by the government were required to be fully equipped with essential medicines and to implement zero-margin sales.[6]

However, unnecessary or inappropriate use of medicine threatens people’s lives and health and can also drain social resources and exacerbate drug resistance.[7] Typically, with the widespread use and abuse of antibiotics, the problem of bacterial resistance has become a focus of global concern.[8, 9] Currently, the abuse of antibiotics in China is a common problem.[10, 11] The rate of antibiotic prescriptions for both inpatients and outpatients is high in China.[11] A survey showed that antibiotics were included in 52.9% of the outpatient visit prescription records and 77.5% of the inpatients received antibiotic therapy among 48 primary health care facilities in China.[12] Regarding inappropriate use of antibiotics in children, studies have indicated that 20%-50% antibiotic use for children was unnecessary.[13] As early as 2001, the WHO developed a Global Strategy for the Containment of Antimicrobial Resistance and provided an intervention framework for delaying the emergence of drug-resistant bacteria and reducing its spread.[14] In China, since July 1, 2004, the China Food and Drug Administration (CFDA) issued a regulation that residents can only buy antibiotics with a prescription; in May 2012, China promulgated “management measures for the clinical use of antibacterial drugs” to standardize antibiotic use in hospitals.[15, 16]

Although many studies have demonstrated the effects of Chinese health reform on medicine accessibility by measuring the affordability and distribution of essential medicines in health facilities, seldom has a study explored the level of medicine access and use from the prospective of Chinese residents.[17] Keeping in mind the background described above, this study viewed a family as a unit and used the household survey method recommended by the WHO to investigate the medicine use of residents and to explore the potential factors influencing household medicine use.[18, 19] The influencing factors of care-seeking outside the home and the determinant factors influencing the use of antibiotics in household members with acute condition are analyzed in this paper.

## Materials and methods

### Ethics statement

The study was approved by the Ethics Committee of Sichuan University West China Medical Center. The Health and Family Planning Commission of Sichuan Province approved the study before the data collection began. The participants were informed of the our study aims prior to their participation. All participants provided signed written informed consent.

### Survey methodology

In 2008, WHO developed a standardized rapid cluster sample survey method to evaluate the accessibility and use of medicines in households in middle- and low-income countries.[18, 19] A structured questionnaire in the manual for the Household Survey to Measure Access and Use of Medicines was primarily used to collect data from the youngest household member who had experienced an acute illness within the previous 2 weeks and from the oldest household member with a chronic disease. In either case, the prescription, source and cost of every medicine taken during the illness were queried. The questionnaire also collected data on the household demographics and socioeconomic characteristics, as well as the behaviors, opinions, and experiences of people confronted with illness.[19] Referring to the WHO questionnaire and considering the actual status of household medicine use in China, we adjusted some items from the original WHO questionnaire to suit the practical background and purpose of our research.

Sichuan Province is located in southwest China and has 21 cities; Chengdu is the capital. Sichuan Province had a population of approximately 81 million people in 2014, ranking fourth among the 34 provinces, municipalities and autonomous regions in China, and ranking first among the 12 western provinces in China.[20] The GDP per capita of Sichuan Province in 2014 was 5719 dollars, placing it in the middle to lower income level in China, and ranking it 7th among the 12 western provinces.[20] Sichuan Province is broadly representative of the typical health and health system status of the 12 western provinces of China. Thus, we selected it as a representative population.

Concerning the method of selecting a survey area recommended by WHO and considering the actual situation (e.g., regionally available levels of medical service, technology and treatment, actual distribution of the population and medical institutions) of Sichuan Province, we used a stratified multistage random cluster sampling method to conduct this survey. First, according to the GDP per capita of the cities in Sichuan Province in 2014, we selected the capital city (Chengdu) and the city with the lowest per capita GDP (Bazhong). In decreasing order, the remaining four cities, Mianyang, Neijiang, Guang’an and Nanchong, were selected through systematic sampling, based on their respective per capita GDP. Secondly, we selected two urban areas near each city center and two suburban areas (one close to urban and one far from urban) in each selected city area, and then selected two rural areas (one close to and one far from each suburban) in each selected suburban area. Finally, we selected two primary sampling units(communities or villages) from each selected urban, suburban areas and two selected rural areas, and then we randomly sampled 16–17 households in each primary sampling unit to compose a sample of 1200 total households. The sample was designed to estimate the key percentage indicators in the survey sample, with a 95% confidence interval of approximately ±6%.[19]

### Data collection

After the data collectors were trained and the survey preparation was completed, the household survey was performed from March to July 2015. The data collectors were allocated into groups of two, and each group entered the reserved household with the help of community officers or staff and conducted a face-to-face questionnaire interview with the head of household under consent. To be surveyed, the head of household was required to 1) be the main health care decision-maker or designated household caregiver and 2) be knowledgeable about the health statuses of the household members, as well as the household health utilization and household health expenditures. The respondents’ participation was voluntary, and they agreed to complete the questionnaire as honestly as possible.

After the household survey was completed, open source software EpiData Entry v·3·1 (the EpiData Association, Denmark) was used for data entry. Two people were responsible for entering and checking the data through double data entry.

### Study variables

The analysis in this study primarily focused on the factors that determine care-seeking outside the home and subsequent antibiotic use by the household members who had suffered an acute illness within the previous 2 weeks. The outcome variable of seeking care outside the home was an important method of receiving antibiotics treatments, and it was a primary method of receiving medication and other health care for household members with acute illnesses. Therefore, in this paper, analyzing the influencing factors of seeking care outside the home was used as a basic measure of the household members’ access to medicines, and the predictor of antibiotic use when seeking care outside the home was analyzed to evaluate the residents’ antibiotic use.

In this paper, the variable “low-income households” is defined as a household having a surveyed per capita income in the lowest income quintile, according to data from the Per Capita Income of Rural and Urban Households by Income Quintile (2014) for Sichuan Province.[21]

### Analysis

Data were analyzed with SPSS software, version 20.0(IBM) using descriptive statistics and multivariate logistic regression analysis. For the multivariate logistic regression, bivariate analyses were first performed to identify the variables associated with the two outcome variables, and variables found to be associated with the outcome variables with p<0.10 in the bivariate analyses were screened out. We also selected variables with p>0.10 in the bivariate analyses, but these variables may be associated with the outcome variables in practice, based on our practical experience. Finally, the two above mentioned types of variables were retained in the final multivariate models. Odds ratio (OR) estimates are provided, with 95% confidence intervals (95%CI). The potential predictor variables of the two outcome variables included household-level factors (socioeconomic status, distance from health care facilities, respondents’ education, respondents’ opinions on local health care), patient-level factors (sex, age, reported symptoms, perceived severity of symptoms) and health service-level factors (types of health care services sought).

## Results

Considering the possibility of invalid questionnaires, we surveyed approximately 1200 households, and after the invalid questionnaires were eliminated, 1103 valid questionnaires were obtained. There were 458 households that reported having had at least one acute illness in the previous 2 weeks, and there were 164, 123 and 171 households reporting acute illness in urban areas, suburban areas and rural areas, respectively (Table 1).

**Table 1:** Characteristics of households with acute conditions and results of the bivariate analysis of the two outcome variables.

Categories	Urban areas	Suburban areas	Rural areas	Total
The total number of surveyed households	380	341	382	1103
Number of households with acute conditions	164	123	171	458
Proportion of households with children (%)	57.9	68.3	59.1	61.1
Age of children (%)				
Younger than 5 years	58.9	48.8	58.4	55.7
5–15 years	54.7	57.1	67.3	60.0
Traveled to the nearest health facility within 15 minutes (%)	81.7	95.9	79.5	84.7
Low income (%)	34.8	38.2	28.1	33.2
Responses by head of household				
Age (%)				
Under 44	29.9	26.8	35.7	31.2
45–59 years	34.1	27.7	36.8	33.4
60 and over	36.0	45.5	27.5	35.4
Educational level (%)				
Middle school or less	55.5	61.0	76.6	64.9 ^a^
High school (vocational school)	18.9	20.3	12.9	17.0
Undergraduate (college) and above	25.6	18.7	10.5	18.1
Perceptions (%)				
The opening hours of their public health care facilities were convenient	79.9	82.1	86.0	82.8
Medicines were usually available at their public health care facilities	91.5	91.9	89.5	90.8
Medicines were usually available at their retail pharmacies	92.7	95.1	88.9	91.9
Medicines at the public health care facility were less expensive than medicines at a retail pharmacy	69.5	78.0	67.3	71.0 ^e^
Can usually afford all costs of the needed medicines	95.1	92.7	90.6	92.8
The quality of services at their public health care facilities was good	77.4	76.4	86.5	80.6

Seeking care outside the home: ^a^ P<0.1, ^b^ P<0.05, ^c^ P<0.01 received antibiotics when seeking care outside the home: ^d^ P<0.1, ^e^ P<0.05, ^f^ P<0.01

### Characteristics of the surveyed households reporting acute illness

As a whole, 61.1% of the households reporting acute illness had at least one child. Approximately one-third of 458 households were low income, and the proportion of low-income households was the highest in the suburban areas (38.2%). In contrast, the proportion of low-income residents was lowest in the rural areas (28.1%). The number of heads of household younger than 45 years of age, 45–60 years, and older than 60 years of age were similar. Among the heads of household, 64.9% had a middle school or less education level, and the proportion was highest in the rural areas (76.6%).

Of the 458 households, 84.7% reported that they had traveled to the nearest health facility within 15 minutes. The proportion that reported having traveled to a health facility within 15 minutes was noticeably higher in the suburban areas compared with the urban or rural areas, and the differences among these households (i.e., suburban, urban and rural) were statistically significant, according to the chi-squared test (χ^2^ = 16.652, P<0.001). But as we surveyed, the transportation approaches to health facilities used by households in urban, suburban and rural areas are different(i.e. by walk, riding, bus or car), so the distance of traveling time of 15 minutes to health facilities in different areas are some different.

More than 90% of the heads of household agreed that medicines were usually available at their public health care facilities and retail pharmacies, and 82.8% agreed that the opening hours of their public health care facilities were convenient. Most of the heads of household (80.6%) agreed that the quality of services at their public health care facilities was good. Most respondents living rural areas agreed with this statement (86.5%), and this percentage was clearly higher than that in urban areas (77.4%) and suburban areas (76.4%) according to the chi-squared test (χ^2^ = 6.284, P<0.05). A total of 92.8% of the heads of household thought that they could usually afford the costs of the medicines that were needed (Table 1).

### Characteristics of individuals with acute conditions

Most acute illnesses occurred in women (55.5%). In total, 41% of the individuals with acute illness were children, and the proportion occurring in children younger than 5 years of age (24.2%) was larger than that in any other age group of the remaining children. Upper respiratory (UR), fever and gastrointestinal symptoms were the most frequent symptoms, occurring in 85.4%, 23.8% and 9.4% of cases, respectively. The acute illnesses were described as not severe in 84.5% of individuals. Most individuals (97.2%) sought care outside their home (approximately one-half selected a public health care facility, 20.4% selected a private hospital, and 29.4% selected a retail pharmacy). Most of the sick people (97.4%) took medicine when they had an acute condition.

Overall, only 15.9% of the individuals with acute conditions reported that the medical insurance reimbursement covered at least one medicine. The proportion reporting reimbursement in the rural areas (25.7%) was noticeably higher than that in the urban areas (12%) and suburban areas (7.4%). Moreover, the differences among the households in the three areas were statistically significant, according to the chi-squared test (χ^2^ = 20.922, P<0.001). The average out-of-pocket expenses for medicines per acute patient accounted for 3.1% of the total household expenditure in that month, and the average out-of-pocket expenditure for medicines per acute patient was highest in the urban areas (19.4±4.0 dollars) and lowest in the rural areas (16.0±7.0 dollars). Most individuals with acute conditions (81.4%) kept their medicines at home, and among the three levels of households, those in the suburban areas (89.4%) were more likely to keep their medicines at home, while only 71.9% in rural areas did so (Table 2).

**Table 2:** Characteristics of the individuals with acute conditions and the results of the bivariate analysis of the two outcome variables.

Categories	Urban areas	Suburban areas	Rural areas	Total
Number of households with acute conditions	164	123	171	458
Sex (%)				
Male	45.1	44.7	43.9	44.5 ^b^
Female	54.9	55.3	56.1	55.5
Age (%)				
Younger than 5 years	23.2	22.8	26.3	24.2
5–15 years	14.0	22.0	15.8	16.8
15 years and older	62.8	55.2	57.9	59.0
Reported symptoms (%)				
Upper respiratory (cough, runny nose, sore throat, earache)	82.9	87.8	86.0	85.4 ^f^
Difficulty breathing, fast breathing	0.6	0.0	0.0	0.2
Fever (fever, headache, hot body)	25.6	22.0	23.4	23.8
Gastrointestinal (diarrhea, vomiting, nausea, could not eat)	11.0	6.5	9.9	9.4
All other symptoms	4.3	3.3	1.8	3.1
Perceived acute illness severity (%)				
Very severe	2.4	2.4	2.9	2.6
Somewhat severe	12.2	17.9	9.9	12.9
Not severe	85.4	79.7	87.2	84.5
Sought care outside the home (%)	95.7	98.4	97.7	97.2
Received care at (%)				
Public health care facility	43.3	41.3	61.7	49.7 ^d^
Private hospital	23.6	24.8	14.4	20.4
Retail pharmacy	33.1	33.1	23.3	29.4
Others	0.0	0.8	0.6	0.5
Took medicines	96.3	98.4	97.7	97.4
Took antibiotics (%)	39.9	38.0	41.9	40.1
Health insurance covers at least one medicine (%)	12.0	7.4	25.7	15.9
Average out-of-pocket expenditure for medicines per acute patient (dollars, 95% CI) *	19.4±4.0	17.9±3.8	16.0±7.0	17.8±5.2
Average out-of-pocket expenditure for medicines per acute patient accounted for total monthly household expenditure (%)	3.1	3.1	3.1	3.1
Had medicines at home (%)	85.4	89.4	71.9	81.4

Seeking care outside the home: ^a^ P<0.1, ^b^ P<0.05, ^c^ P<0.01 received antibiotics when seeking care outside the home: ^d^ P<0.1, ^e^ P<0.05, ^f^ P<0.01

*: Exchange rate of RMB against the U.S. Dollar was based on the annual rate of RMB against the U.S. dollar in 2015 from the State Statistical Bureau in China.

### Antibiotic treatment

A total of 40.1% of individuals who took medicine while sick received antibiotics. We found that beta lactam (87.2%) and macrolides (13.4%) were the most commonly used antibiotic classes among antibiotic users. Of the beta lactams, amoxicillin was prescribed most-often (52.3%), followed by cephalosporins (42.4%). Approximately 20% of antibiotics users received combination antibiotics. Most antibiotic use (68.5%) was recommended by doctors, and approximately a quarter of the antibiotic use (24.3%) was requested by patients. Approximately one-half of those using antibiotics (47.7%) received them from a public health care facility, followed by retail pharmacies (33.2%) and private hospitals (19.3%). In addition, in rural areas, the proportions of those using antibiotics obtained from retail pharmacies and private hospitals were nearly identical (Table 3).

**Table 3:** Sources and types of antibiotics received.

Categories	Urban areas	Suburban areas	Rural areas	Total
Number of individuals treated with antibiotics	63	46	70	179
Types of antibiotics (%)				
Beta lactams	88.1	79.5	91.3	87.2
Amoxicillin	49.2	47.7	58.0	52.3
Cephalosporins	47.5	36.4	42.0	42.4
Aminoglycoside	1.7	0.0	1.4	1.2
Macrolides	13.6	22.7	7.2	13.4
Quinolones	3.4	6.8	2.9	4.1
Others	6.8	4.5	1.4	4.1
Combination antibiotics (%)	18.6	20.5	21.7	20.3
Antibiotics were recommended by (%)				
Doctors	68.1	62.0	74.6	68.5
Neighbors, friends	1.4	0.0	3.4	1.7
Other household members	6.9	8.0	1.7	5.5
Patients themselves	23.6	30.0	20.3	24.3
Antibiotics were obtained from (%)				
Public health care facility	48.6	44.0	49.2	47.5
Private hospital	16.7	16.0	25.4	19.3
Retail pharmacy	34.7	40.0	25.4	33.2

### Predictors of seeking care outside the home

According to the bivariate correlation analysis (Tables 1 and 2), and considering the actual situation in practical experiences, 7 variables for care-seeking outside the home among individuals with an acute condition were brought into the final multivariable logistic regression model. In Tables 1 and 2, the educational level of the heads of household (P<0.1) and the sex of the individuals with acute conditions (P<0.05) were associated with seeking care outside the home among individuals. In Table 4, compared with men, we found that women were less likely to seek care outside the home when suffering from acute illness (OR: 0.20, 95%CL: 0.04–0.96, P = 0.044).

**Table 4:** Multivariate determinants of seeking care outside the home to treat an acute illness.

Variables	OR	95%CL	P-value
Household-related predictors			
Low income (vs no)	1.07	0.32–3.60	0.908
Traveling to the nearest health facility within 15 minutes (vs >15 min)	0.67	0.13–3.41	0.632
Educational level of the head of household (vs middle school and less)			
High school (vocational school)	-	-	0.997
Undergraduate (college) and above	3.71	0.42–32.58	0.236
Patient-related predictors			
Female (vs male)	0.20	0.04–0.96	0.044
Age (vs 15 years and older)			
Younger than 5 years	0.56	0.13–2.38	0.432
5–15 years	0.76	0.15–3.85	0.738
Reported symptoms (vs no)			
Upper respiratory (cough, runny nose, sore throat, earache)	3.49	0.50–24.56	0.209
Difficulty breathing, fast breathing	-	-	1.000
Fever (fever, headache, hot body)	5.23	0.50–54.29	0.166
Gastrointestinal (diarrhea, vomiting, nausea, unable to eat)	1.01	0.11–8.90	0.996
The perceived severity of the acute illness (vs not severe)			
Very severe	-	-	0.999
Somewhat severe	2.75	0.30–25.01	0.369

-: indicates infinity or near zero.

### Predictors of antibiotic use when seeking care outside the home

For antibiotic use when seeking care outside the home, 10 variables were brought into the final multivariable logistic regression model. As shown in Tables 1 and 2, the following variables were associated with antibiotic use when seeking care outside the home: the opinions of the heads of household (such as agreeing that the medicines from a public health care facility were less expensive than those from a retail pharmacy [P<0.05]), symptoms of acute illness (P<0.01) and the types of health care facilities that were sought (P<0.1). As shown in Table 5, we found that the symptoms of acute illness were strong predictors of antibiotic use when patients sought care outside the home. Overall, individuals with acute conditions were more likely to receive antibiotics if they suffered from fever (OR: 2.51, 95%CL: 1.50–4.20, P = 0.001) or UR symptoms (OR: 2.03, 95%CL: 1.02–4.03, P = 0.043). The odds of taking antibiotics also depended on the source of care and the patient’s age. Compared with those who bought medicines from a retail pharmacy, patients visiting a private hospital were more likely to receive antibiotics (OR: 2.20, 95%CL:1.20–4.05, P = 0.011). Patients younger than 5 years of age were less likely to receive antibiotics compared with patients who were 15 years of age or older (OR: 0.52, 95%CL: 0.30–0.91, P = 0.021). Moreover, patients from low-income households showed less of a tendency to receive antibiotics (OR: 0.61, 95%CL: 0.38–0.95, P = 0.031), and patients who agreed that medicines from a public health care facility were less expensive than those from a retail pharmacy were more likely to receive antibiotics (OR: 1.64, 95%CL: 1.07–2.50, P = 0.023).

**Table 5:** Multivariate predictors of antibiotic use to treat acute illness.

Variables	OR	95%CL	P-value
Household-related predictors			
Low income (vs no)	0.61	0.38–0.95	0.031
Traveling to the nearest health facility within 15 minutes (vs >15 min)	0.80	0.45–1.41	0.437
Educational level of the head of household (vs middle school and less)			
High school (vocational school)	1.18	0.68–2.07	0.553
Undergraduate (college) and above	1.46	0.83–2.54	0.187
Perceptions of the head of household			
Medicines were usually available at their retail pharmacy (vs no or unsure)	1.77	0.80–3.95	0.161
Medicines in public health care facility were less expensive than medicines in retail pharmacy (vs no or unsure)	1.64	1.07–2.50	0.023
Patient-related predictors			
Female (vs male)	1.05	0.70–1.57	0.830
Age (vs 15 years and older)			
Younger than 5 years	0.52	0.30–0.91	0.021
5–15 years	0.85	0.49–1.49	0.564
Reported symptoms (vs no)			
Upper respiratory (cough, runny nose, sore throat, earache)	2.03	1.02–4.03	0.043
Difficulty breathing, fast breathing	-	-	1.000
Fever (fever, headache, hot body)	2.51	1.50–4.20	0.001
Gastrointestinal (diarrhea, vomiting, nausea, unable to eat)	0.95	0.41–2.19	0.897
Perceived acute illness severity (vs not severe)			
Very severe	0.64	0.18–2.32	0.494
Somewhat severe	1.02	0.56–1.89	0.939
Source of the care sought during the illness (vs retail pharmacy)			
Public health care facility	1.52	0.92–2.53	0.103
Private hospital	2.20	1.20–4.05	0.011

-: indicates infinity or near zero

## Discussion

The study results showed that the accessibility of medicines for acute illness among households in western China was favorable. Overall, 97.4% of patients took medicines to treat acute illnesses. The high accessibility of medicines was demonstrated by the following findings: in terms of the geographical location of medical institutions, 84.7% of the households traveled to the nearest health facility within 15 minutes; in terms of the availability of medicines, more than 90% of the heads of household agreed that medicines were usually available at their public health care facilities and retail pharmacies, and 82.8% demonstrated positive attitudes about the opening hours of their public health care facilities. In terms of the affordability of medicines, the average out-of-pocket payment for medicines per acute patient accounted for 3.1% of the total monthly household expenditures, and most of the heads of household said that they could usually afford all costs of the needed medicines. These findings can be used as the practical evidences for evaluating the effects of Chinese new health-care reform by government.

Our findings showed that the medical insurance reimbursement system in western China still needs to be improved. Only 15.9% of patients reported that medical insurance reimbursement covered at least one medicine, and this proportion was notably higher in rural areas than in urban and suburban areas. These proportions may be attributed to the Medical Insurance System in China. In fact, the medical insurance plans for urban employers and urban residents and the New Medical Cooperative Scheme(NMCS) were the three basic medical insurance schemes in China, and there were some clear differences in the proportion and scopes of the three medical insurance reimbursements.[22] Therefore, we suggest that the government should continue to increase its financial support in health care and stress rational allocations of medical resources in urban and rural areas. Moreover, the government should also further improve the structure of the medical security system and gradually expand the scope and coverage of medical insurance reimbursement.

Our results showed that the use of antibiotics must be further rationalized in western China. Among the patients with acute conditions who sought care outside the home, 40.1% received antibiotics. This level of antibiotic use represented a clear decrease compared with the statistics reported in other Chinese studies.[12, 23] However, the rate of antibiotic use was still higher than the WHO recommendation of 30%.[24] China still needs to make a considerable effort to catch up with the antibiotic use rate in developed countries. The findings that 47.7% of the antibiotics use was prescribed by public health care facilities, and 68.5% was recommended by doctors indicated that most of the antibiotic use in patients could be influenced by medical providers. Moreover, we found that the broad-spectrum antibiotics amoxicillin (52.3%) and cephalosporins (42.4%) were the antibiotics most often used. In fact, in some cases, health care providers relied too heavily on the therapeutic efficacy of broad-spectrum antibiotics, and as a result, were unable to appropriately treat the case and abuse broad-spectrum antibiotics. Abuse of antibiotics was also closely related to the lack of public awareness of antibiotics. Our results showed that approximately a quarter of the antibiotics used were requested by patients, which suggested that the public lacked a correct understanding of antibiotics. Our results corroborated the findings of a report about the situation of medication public safety from the China Food and Drug Administration. The report showed that 20.7% of residents and 35.2% of netizens agreed that antibiotics are equivalent to anti-inflammatory medicines.[25] In the future, health departments in China should improve the national medicines policy to strengthen the standardized management of key antibiotics and the supervision and standardization of the medication behaviors of health care providers. At the same time, health departments should also enhance public information campaigns about the rational use of antibiotics among community residents.

Fever and UR symptoms were strong predictors of antibiotic use in our results. This indicated that the concept “cold symptoms require the use of antibiotics” commonly believed by doctors still existed. Furthermore, when being treated outside the home, patients younger than 5 years of age were less likely to receive antibiotics compared with patients older than 15 years old, which indicates that the use of antibiotics in children tends to be standardized in west China. Interestingly, our results showed that low-income patients were less likely to be treated with antibiotics when seeking care outside the home, which was contrary to findings from European countries, in which high-income groups were less likely to use antibiotics.[26] This discrepancy indicated that there was a great difference in the public conception of antibiotic use between the residents of developing countries and those in developed countries.

In contrast to an African study, our study found that women were less likely than men to seek care outside the home when suffering from acute illness,[27] which suggests that different sex groups tend to take different actions when faced with illness. There are more ORs close to infinity or zero in Table 4 because in our survey, few people with acute conditions did not seek care outside the home.

Although the rapid cluster sample survey methodology recommended by WHO to evaluate the accessibility and use of medicines in countries or cities with limited resources did provide some meaningful data with a small sample and an easy-to-administer instrument, this study also has some limitations. At present, many communities in China are classified by work units or income levels, therefore, it is difficult to select a diverse group of households to survey by sampling. Because of the large population in Sichuan Province, there are great differences in the socioeconomic levels of the cities, as well as research funding and human resource limitations; therefore, the small sample size recommended by WHO may not cover remote or minority areas and may have resulted in an underestimated variance. Moreover, although the data collectors underwent rigorous training and supervision owing to individual defense mechanisms and the fact that household income is a relatively sensitive issue in China, the household income information collected by the questionnaire may not reflect precise income levels. Seasonal fluctuations in morbidity affect the need for medicines, and we cannot determine how such fluctuations may have influenced our results because we have no reliable estimates of the incidence of common acute illnesses in the surveyed cities.[27] Considering the hierarchical characteristics of the samples (i.e., suburban, urban and rural), multi level regression model would be more suitable in the study. But because the sample size of each level households was not enough, we just conducted the logistic regression analysis for the total levels in this study, instead of for the 3 different levels individually.

Despite the above limitations, our study provides empirical evidence of the use of medicines among residents in China for the first time, and it has achieved the primary goal of determining antibiotic use. This study can help the Chinese government or other countries to better understand the current situation regarding the use of medicines and care services among residents of western China. The findings can also be used as a baseline for the future to measure the changes of antibiotic use over time or to evaluate the impact of interventions to reduce antibiotic use. Next, we will conduct this study in other provinces in China to fully understand the current situation regarding the use of medicines and care services among Chinese residents.

## Conclusion

The accessibility of medicines for acute illness is favorable among households in western China. However, the medical insurance reimbursement system needs improvement. The nature of symptoms and care-seeking patterns had the greatest influence on the decisions to take antibiotics among residents with acute conditions. The percentage of antibiotic use in patients with acute illness has declined, but the indications for the use of antibiotics must still be standardized.

## Supporting information

S1 Protocol Household survey protocol of measuring access and use of medicines among residents in Sichuan Province, China (in English). (DOCX)

S2 Protocol Household survey protocol of measuring access and use of medicines among residents in Sichuan Province, China (in Chinese). (DOCX)

S1 Questionnaire Questionnaire for the Household survey to measure accessibility and use of medicines in Sichuan Province, China. (DOCX)
