# Supplementary material for: Access to medicines for acute illness and antibiotic use in residents: A medicines household survey in Sichuan Province, western China
Source: PLoS One. 2018 Aug 16;13(8):e0201349. doi: 10.1371/journal.pone.0201349 (PMC6095499; doi:10.1371/journal.pone.0201349)
Supplement: S1 Protocol — (DOCX) [file pone.0201349.s001.docx]

**Household Survey Protocol of Measuring Access and Use of Medicines among Residents in Sichuan Province, China**

**Research institute:** Pharmaceutical Policy & Pharmacoeconomics Research Center, West China School of Pharmacy, Sichuan University

**Cooperative institute:** Health and Family Planning Commission of Sichuan Province

**Research background**

In March 2009, China started a new round of health care system reform, in December of the same year, ten government departments in Sichuan province released the document of *Opinions on Implementing the National Essential Medicines System* *(NEMS)* in Sichuan. In June 2012, the General Office of the State Council released the document of *Opinions on Pilot Reform of County-level Public Hospitals*, which marked the implementation of the Zero Mark-up Medicines Policy. Correspondingly the Health and Family Planning Commission of Sichuan Province confirmed this policy and released a list of public hospitals in all 21 cities of Sichuan to pilot implement the Zero Mark-up Medicines Policy.

Along with the implementation of multiple policies of new medical reform, the accessibility of essential medicines and the rationality of medicine use were reported improved. However, the people's feelings about the effect of reform are unclear, and the actual accessibility to medicines from the view of residents needs to be access. So we conduct this household survey to measure the accessibility and use of medicines among residents in Sichuan province in order to provide empirical data to support for improving China's medical reform and drug policy in the future.

**1. Research purposes**

1) To evaluate the accessibility of medicines for acute and chronic diseases, the rational use of antibiotics, the storage status of household medicines, and medicine economic burdens of residents in Sichuan province, China.

2) To identify the influencing factors of access to medicines for acute and chronic diseases and the primary determinant factors that influence the use of antibiotics. Therefore to provide empirical data to support for improving China's medical reform and drug policy in the future.

**2. Research methods and pathway**

**2.1 Survey objectives and Sampling method**

The *WHO Operational Package for Assessing, Monitoring and Evaluating Country Pharmaceutical Situation* recommends to select 5 regions to conduct the survey. It suggests to select the largest or capital city, and the most rural or lowest income-generating area firstly, and then to randomly select three other geographical areas from the remaining regions. The *WHO’s Manual for the Household Survey to Measure Access and Use of Medicines* recommends that households are selected around each reference health care facility with <5 km, 5-10 km or >10 km.

Considering the actual situation in China and the actual distribution of households of Sichuan province(e.g., regionally available levels of medical service, technology and treatment, actual distribution of the population and medical institutions), it is not suitable to select such representative households in Sichuan province in terms of the WHO tool manual. So, a stratified multi-stage random cluster sampling method is adopted to select households.

In according to the WHO’ manual, 180 households of each sites and a total of 1080 households in a research area should be sampled. Considering there may be some invalid questionnaires, we increase the sample size by about 10% up to 1,200 households.

**2.2 Sampling of the households**

The stratified multistage random cluster sampling steps list as following:

**2.2.1 Select the survey areas**

Set city or state as sampling unit: 6 cities (states) out of the all 21 cities are sampled equidistantly according to the rankings of per capita GDP of Sichuan Province in 2014(Table 1). There are: Chengdu(capital city), Mianyang, Neijiang, Guang'an, Nanchong, Bazhong) .(Table 1 )

**Table 1. The sample city distribution**

| **Regions** | **GDP Per capita 2014** |
| --- | --- |
| **Panzhihua** | **70611.37** |
| **Chengdu** | **70337.61** |
| **Deyang** | **42939.91** |
| **Zigong** | **39199.50** |
| **Leshan** | **37092.7** |
| **Mianyang** | **33784.32** |
| **Ziyang** | **33478.94** |
| **Yibin** | **32336.17** |
| **Meishan** | **31724.75** |
| **Neijiang** | **31111.86** |
| **Ya’an** | **30149.96** |
| **Luzhou** | **29670.03** |
| **Liangshan** | **28665.21** |
| **Guan’an** | **28521.23** |
| **A’ba** | **27161.02** |
| **Suining** | **24719.08** |
| **Dazhou** | **24449.10** |
| **Nanchong** | **22669.31** |
| **Guangyang** | **22247.15** |
| **Ganzi** | **18176.31** |
| **Bazhong** | **13791.37** |
| **Average** | **34435.195** |

**2.2.2 Select the survey districts, counties and townships**

Set district, county and township as sampling unit: 2 districts, 2 counties，and 4 townships are selected from each selected city. The 2 districts are near the city center, and the 2 counties are selected according to the geographical location (one close to district and one far from district). Then, select 2 townships from each selected county (one close to and one far from each selected county). Finally, 12 districts, 12 counties and 24 townships are sampled in six selected cities. The sample districts, counties and townships are shown in Table 2.

**Table2. The lists of districts, counties and townships to be surveyed in Sichuan, China**

| Province | City | District | County | Township |
| --- | --- | --- | --- | --- |
| Sichuan | Chengdu | Wuhou, Jinjiang | Shuangliu, Pixian | Xi hanggang, Dongsheng; Xipu, San daoyan |
|  | Mianyang | Peicheng, Youxian | Jiangyou, Pingwu | Sanhe, Taiping; Nanba, Pingtong |
|  | Neijiang | Shizhong, Dongxing | Weiyuan, Longchang | Gaoshi, Zhenxi; Xiangshi, Longshi |
|  | Guangan | Guang'an, Qianfeng | Yuechi, Wusheng | Jiulong, Shiya; Mengshan, Baiping |
|  | Nanchong | Shunqing, Gaoba | Yingshan, Xichong | Luoshi, Huilong; Gulou, Taiping |
|  | Bazhong | Bazhou, Enyang | Pingchong, Tongjiang | Baiyi, Sima; Guangna, Minsheng |

**2.2.3 Select the survey households**

Set households as sampling unit. 4 representative communities or villages are selected from the selected districts, counties and townships according to the actual situation(e.g., the populations, economic situations, types of household); then, 16-17 households are selected by isometric sampling in each community or village according to the list of permanent residents provided by the local neighborhood committee. The isometric sampling steps are as following:

(1) Prepare a list of permanent households: Number the households according to the list of permanent residents in the sample communities or villages provided by the neighborhood committee.

(2) Determine the sampling interval based on the number of households in the sample communities or villages provided by the neighborhood committee:

Sampling interval = number of households in the sample community (village) / 16-17(rounding and taking the round numbers)

(3) Identify the sampling households

i. Determine the first household to be drawn: Take one RMB bill at random and get the last four digits from the bill number; then, the number is divided by the sampling interval and the remainder of it is determined as the K value (if the remainder is 0, the K value is the sampling interval); finally, the K value is determined as the first household number to be sampled.

ii. Determine the remaining sampled households: The second household number to be drawn is the number of the K value adding the sampling interval, and so forth. (if the sampled household is absent or refuses to accept this survey, select the household adjacent to the sampled household which have not been drawn as a alternative household.)

**2.3 Select the survey head of the household**

The survey will be conducted to the head of the selected households. The head of the households should meet the following criteria:

1) Main health care decision maker；

2) Be most knowledgeable about health of household members and health expenditures of the household；

3) Be most knowledgeable about medicines use of sick household members.

**2.4 Design questionnaire**

Referring to the structured questionnaire in *the Manual for the Household Survey to Measure Access and Use of Medicines* recommended by WHO, adjust some items from the original WHO questionnaire to suit the practical background and purpose of this research. The main content are as following:

1. The primary information of households, including: households demographic information, households economic, health expenditure and daily consumption of households and so on;
2. The information about medicine use of the youngest household member with an acute illness within the previous 2 weeks and the oldest household member with a chronic disease, including: the prescription, source and cost of every medicine taken, medical insurance and so on;
3. The information about reserved medicines of households;
4. The opinions of the head of the household about the affordability and availability of medicines, the medicines quality, the medical service quality and so on.

**2.5 Implement survey**

The research is assisted and arranged by the Health and Family Planning Commission of Sichuan Province and the local Health and Family Planning Commissions(Health Bureaus).

The data collectors are composed of undergraduate and graduate students from West China School of Pharmacy, Sichuan University. The data collectors are trained by research leader before the survey and are allocated into groups of two.

After contact the heads of households under the assistance of local community staff, and get verbal informed consent of them, groups enter the households and conduct a face-to-face questionnaire interview with the head of the household.

The survey is carried out by a way of voluntary and anonymous interview and the questionnaire does not involve the name, contact information, home address and other important privacy information of the respondents.

The research period set from April to June 2015, and the specific research arrangements are as follows (it may be adjusted according to the actual progress of the survey):

April 16th to April 24th: Chengdu.

April 27th to May 8th: Mianyang.

May 12th to May 20th: Neijiang.

May 21st to May 31st: Nanchong.

June 2nd to June 12th: Guang'an.

June 16th to June 26th: Bazhong.

**2.6 Data quality control**

(1) Pre-survey quality control

Before the survey, the researcher leader conduct a unified training for the data collectors, the training content includes the interpretation of each question in the questionnaire, the requirements for filling in, the method of asking, etc. Then, conduct a preliminary survey to find the problems in the actual survey and propose solutions to solve it.

(2) Quality control during the survey

The data collectors are allocated into groups of two in order to avoid the personal bias understanding in the questionnaire interview. In order to improve the coordination of the head of the sampled household, we provide nail scissors as survey gifts for the participation of each household.

(3) Post-survey quality control

When finishing every day’s household survey, the research leader sorts out and checks the questionnaires collected on the day. For the non-standard records, the research leader should point it out and let him/she pays attention on it when next interview; for the ambiguous answers in the questionnaires, the research leader should confirm to the data collector for this information to ensure the accuracy of collected data.

**3. Data analysis**

After the household survey is completed, the EpiData software is used for data entry. Two people are responsible for entering and checking the data through double data entry. The SPSS software is used to do the descriptive statistics and multivariate logistic regression analysis with the all data.

1. **Project support and research funding**

This study is supported by the National Natural Sciences Foundation of China (71473170). It provides funds for survey transportation, travel, labor services, data printing, research gifts, research collaboration and coordination, etc.
