# Supplementary material for: Access to medicines for acute illness and antibiotic use in residents: A medicines household survey in Sichuan Province, western China
Source: PLoS One. 2018 Aug 16;13(8):e0201349. doi: 10.1371/journal.pone.0201349 (PMC6095499; doi:10.1371/journal.pone.0201349)
Supplement: S2 Protocol — (DOCX) [file pone.0201349.s002.docx]

**新医改背景下四川省药品可及性及使用情况入户调查研究方案**

调研单位：四川大学华西药学院药物政策与药物经济学研究中心

协作单位：四川省卫生和计划生育委员会

**研究背景**

2009年3月，我国启动了新一轮的医改，同年12 月起，四川省发改委等十部门发布了《四川省国家基本药物制度实施意见》，在30%的县基层医疗卫生机构和城市社区卫生服务机构实施基本药物制度。2012年6月，国务院办公厅发布文件《关于县级公立医院综合改革试点的意见》，标志着取消药品加成政策在县级公立医院开始实施。四川省卫计委于同年7月确定并发布四川省试点名单，包括成都市、绵阳市在内的20个市（州）的27家县级公立医院开始执行取消“以药补医”试点。

新医改多重政策在四川省实施多年以来，基层医疗卫生机构在基本药物配备和使用、门诊合理用药、降低基本药物价格方面取得阶段性成果，但老百姓对看病医药费用降低的感受并不明显。为此，本文通过对四川省居民家庭药品使用及储备情况进行实证调研，探索新医改多重政策实施对家庭用药产生的影响，为进一步全面评价新医改多重政策实施后对居民家庭药品的可及性、用药行为产生的效果提供另一种参考。

**1、研究目的**

基于WHO家庭用药入户调查指南，采用问卷调查的形式抽样调研四川省居民家庭药品可负担性、可获得性及使用的现状，分析评价我国急慢性病药品可及性、抗生素使用情况，家庭药品储备情况，疾病和药物负担情况，为进一步完善我国医改和药物政策提供参考依据。

**2、研究方法和过程**

**2.1 研究目标与抽样方法**

按照WHO《药品可及性及药品使用情况入户调查指南》，评估一个国家居民药品可及性以及药品使用情况，可按指南推荐标准抽取5个地区展开调查。5个调查地区确定的标准为：首先选择调查范围内最大的地区（省会或是首都），然后选择调查范围内经济最落后的地区，从其余的地区内随机选择其它3个地区。其中，调查家庭的选择是参考调查医疗机构的地理位置，在参考调查医疗机构某个方向上5km以内、5-10km、10km以外分别选取一定数量的调查家庭开展调研。考虑到我国国情以及四川省的医疗卫生水平，医疗机构和人口的实际分布情况，上述WHO的调查家庭选择方法在四川省很难选取到有代表性的样本家庭，因此，本次研究采用分层多阶段随机整群抽样方法。

WHO指南建议每个抽样城市抽样180户家庭，一个调查地区共计抽样1080户家庭。考虑到可能存在的无效问卷，本研究实际调研增加10%左右的样本量，实际抽样1200户家庭。

**2.2 抽样方法**

分层多阶段随机整群抽样方法具体步骤如下：

**2.2.1调查地区的选择**

以市州为抽样单位。参照WHO指南，根据四川省2014年人均GDP排名，等距抽取成都市、绵阳市、内江市、广安市、南充市、巴中市6个市州进行调研,具体抽样城市如表1。

**表1 抽样城市分布**

| 城市 | 2014年人均GDP（元） |
| --- | --- |
| 攀枝花市 | 70611.37 |
| 成都市 | 70337.61 |
| 德阳市 | 42939.91 |
| 自贡市 | 39199.5 |
| 乐山市 | 37092.7 |
| 绵阳市 | 33784.32 |
| 资阳市 | 33478.94 |
| 宜宾市 | 32336.17 |
| 眉山市 | 31724.75 |
| 内江市 | 31111.86 |
| 雅安市 | 30149.96 |
| 泸州市 | 29670.03 |
| 凉山彝族自治州 | 28665.21 |
| 广安市 | 28521.23 |
| 阿坝藏族羌族自治州 | 27161.02 |
| 遂宁市 | 24719.08 |
| 达州市 | 24449.1 |
| 南充市 | 22669.31 |
| 广元市 | 22247.15 |
| 甘孜藏族自治州 | 18176.31 |
| 巴中市 | 13791.37 |
| 平均值 | 34435.195 |

**2.2.2调查城区、县城、乡镇的选择**

以城区、县城和乡镇为抽样单位。从阶段一选取的各市中抽取2个中心城区和2个县城（一个离城区最近，一个离城区最远），并在每个选中的县城中，分别选取2个乡镇（一个离县城最近，一个离县城最远），四川省共计抽样12个城区、12个县城、24个乡镇，具体抽样地区如表2。

**表2 四川省具体调研市、县、乡镇**

| **调查省市** | **调查城市** | **抽样城区** | **抽样县城** | **抽样乡镇** |
| --- | --- | --- | --- | --- |
| 四川省 | 成都市 | 武侯区、锦江区 | 双流县、郫县 | 西航港、东升镇，犀浦镇、三道堰镇 |
|  | 绵阳市 | 涪城区、游仙区 | 江油市、平武县 | 三合镇、太平镇，南坝镇、平通镇 |
|  | 内江市 | 市中区、东兴区 | 威远县、隆昌县 | 高石镇、镇西镇，响石镇、龙市镇 |
|  | 广安市 | 广安区、前锋区 | 岳池县、武胜县 | 九龙镇、石垭镇，猛山乡、白坪乡 |
|  | 南充市 | 顺庆区、高坪区 | 营山县、西充县 | 骆市镇、回龙镇，古楼镇、太平镇 |

**2.2.3 调查家庭的选择**

抽样单位是家庭。在抽样的城区、县城、乡镇中，根据具体实际情况，分别选取4个具有一定代表性的社区或村庄，然后在选取的每个社区或村庄根据居委会提供的常住户名单等距选取16-17户家庭进行问卷调查，四川省共计抽取1200户左右的家庭。

样本住户的等距抽样：

1. 编制常住户清单

根据居委会提供的样本社区或样本村的常住户名单，按名单顺序编号；

1. 根据居委会提供的样本社区或样本村的常住户数，确定抽样间隔

抽样间隔=样本社区（村）常住户数/ 16-17（四舍五入，取整数）；

1. 确定样本住户

①确定第一个应抽住户。随机取一张人民币，取其末四位数，该数除以抽样间隔的余数确定为K值（若余数为0，则K值为抽样间隔），K值则确定为抽样的第一个住户序号

②确定其余抽样住户。K值加抽样间隔为被抽中的第2个住户序号,依此类推。（如果抽样住户外出或不愿意参与，则选取住户相邻未被抽中的住户为备选住户）

**2.3调查户主的选择**

在相关工作人员的协助下，与抽样家庭户主预约好时间，数据收集员按照约定时间进行访问，首先向调查家庭成员解释说明此次调研的目的、方式以及问卷的大致内容，然后询问其是否愿意接受调查，是否对家庭健康状况最清楚、是否是家庭健康方面的主要决策者、是否了解家庭的健康支出情况等问题，如均表示肯定则选择其为调查对象。

**2.4调研问卷设计**

基于WHO《药品可及性及药品使用情况入户调查指南》中推荐的调查项目和问题，结合我国实际情况设计问卷，经过预调查确定调查问卷，调研内容包括：

（1）家庭基本情况：家庭基本人口学信息，家庭经济情况，医疗、药品费用情况，调查户主的基本信息等。

（2）家庭急/慢性病患者用药情况：包括患病类型、患者基本情况、药品费用、药品来源、抗生素使用、医保报销情况等。

（3）家庭储备药品情况：储备药品种类、用途、质量等信息。

（4）医疗卫生服务质量及药品质量认知情况：调查户主对医疗卫生服务及药品的可及性、药品的可负担性、医疗卫生服务及药品的质量认知情况。

**2.5 调研实施**

本次评估由四川省卫生计生委及各地市卫计委（卫生局）协助与安排，在当地社区街道办等工作人员的协助下，按照约定时间对抽样家庭进行访问，在或得受访户主知情同意并自愿配合的前提下，进行面对面访谈式匿名问卷调查，同时发放礼品，当场回收问卷。数据收集人员主要由四川大学华西药学院药物政策与药物经济学研究中心成员，经过前期调研培训和预调查摸底，2人一组实施调研。调研时间从2015年4月至6月。具体调研安排如下(可能根据调研实施情况有所调整)：

4月16日－4月24日，成都市调研

4月27日－5月8日，绵阳市调研

5月12日－5月20日，内江市调研

5月21日－5月31日，南充市调研

6月2日－6月12日，广安市调研

6月16日－6月26日，巴中市调研

**2.6 数据质量控制**

(1)调查前质量控制

在调查前对数据收集员进行统一培训，对问卷过程中每个条目的解释、内容、标准以及调查用语，问询方式进行讲解，对问卷填写的注意事项及规则进行统一说明。

(2)调查中质量控制

为了避免数据收集员对访谈内容可能存在个人理解偏差，问卷调查采取两人一组，与户主进行面对面匿名访谈。在受访过程中，数据收集员要在向调查对象说明此次调研目的和内容，征求得到受访对象同意的情况下开展，为提高户主配合度，确保问卷调查信息真实有效，本次问卷访谈结束后，对每个访谈户主送上精美小礼品。

1. 调查后质量控制

调研每天结束后，数据收集员负责人要对当天收集回来的问卷进行整理与检查，对问卷中存在的不规范记录和内容自相矛盾问题，负责人要向数据收集员进行说明并提出解决方法。

**3、数据处理与统计分析**

回收的调研问卷当天进行有效性评估、剔除无效问卷并进行编号，回收的问卷采用Epidata3.1双人录入并校验，采用SPSS20对居民家庭急慢性病患者药品的可获得性、可负担性和可及性现状，医保报销情况，抗生素使用情况及家庭储备药品情况等进行描述性分析，采用卡方检验、方差分析等对全国及不同省市，城乡不同层次间药品可及性及药品使用的差异进行统计学分析，采用SPSS20软件统计分析模型方法对药品可及性影响因素进行logistic回归分析，得出影响我国居民家庭急慢性病患者药品可及性及抗生素使用的关键因素。

**4、课题支撑及研究经费**

本课题为国家自然科学基金项目《新医改多重政策实施背景下基本药物可及性评价：指标与方法的建立与实证》（项目批准号：71473170）研究内容，由其提供经费用于调研交通、差旅、劳务，资料印制，调研礼品，协作及协调等。
