# Supplementary material for: Access to medicines for acute illness and antibiotic use in residents: A medicines household survey in Sichuan Province, western China
Source: PLoS One. 2018 Aug 16;13(8):e0201349. doi: 10.1371/journal.pone.0201349 (PMC6095499; doi:10.1371/journal.pone.0201349)
Supplement: S1 Questionnaire — (DOCX) [file pone.0201349.s003.docx]

| Interviewer |  |
| --- | --- |
| City, district/county |  |
| Street/township, community/village |  |
| Complete time |  |
| Questionnaire number |  |

Questionnaire for the Household Survey to Measure Accessibility and Use of Medicines in Sichuan Province, China

1. **Basic information of the household**

1.1. How many persons live in this household on a usual day?

1.2. How many children live in this household with the ages are under 15 years old ?

A. under 5 years old B. 5 years old and older

1.3. How many old persons live in this household with the ages are 60 years old and older?

1.4. What is the most common form of transportation for your household members to seek health care? And how much time does it take to reach the following health care facilities or providers?

A. by foot B. by bicycle (electric/motorcycle) C. by car D. by bus

|  | time  （minute） | transportation |  | time（minute） | transportation |
| --- | --- | --- | --- | --- | --- |
| public medical institution |  |  | private medical institution |  |  |
| retail pharmacy |  |  | Shopping mall and supermarket pharmacy |  |  |
| other |  |  |  |  |  |

1.5. Have your household members purchased medical insurance? **［ ］**

A. yes (1.5.1 members purchased medical insurance)

B. no (If the answer is “no”, skip to question 2.1)

1.6. Which medical insurance did the household members purchased medical insurance? 【Multiple choice】 **［ ］**

A. medical insurance for urban employees

B. medical insurance for urban residents

C. new rural cooperative medical insurance

D. supplementary medical insurance

E. commercial medical insurance

F. other

1. **Use of medicines in patients with acute illness**

2.1. Has anyone in the household been ill in the past two weeks with an acute illness? (An acute illness is a condition that appears suddenly and changes rapidly. Upper respiratory infection such as fever, cough, runny nose, gastrointestinal infection such as nausea and vomiting) **［ ］**

A. yes

B. no (If the answer is “no”, skip to the next section of the survey starting at question 3.1)

2.2. How many persons have an acute illness over the past two weeks?

2.3. What is the age of the youngest person who was ill?

2.4. What is the gender of the youngest person who was ill? **［ ］**

A. male B. female

2.5. What type of problems did this person have during the illness? **［ ］**

A. upper respiratory infection (cough, runny nose, sore throat, earache)

B.difficulty breathing, fast breathing

C. fever (fever, headache, fever)

D. gastrointestinal infections (diarrhea, vomiting, nausea, loss of appetite)

E. other

2.6. How serious do you think the illness was? **［ ］**

A. very serious B. more serious

C. generally serious D. nothing serious

2.7. At any point, did this person (or anybody else on his/her behalf) seek care for this illness outside the home? **［ ］**

A. yes B. no (If the answer is “no”, skip to question 2.9)

2.8. From which of the following sources of care did this person receive care at any time during the illness? **［ ］**

A. public medical institution: 1. village clinic; 2. community health service center; 3. township hospital; 4. district and county public hospital; 5. municipal public hospital; 6. above the provincial level public hospital

B. private medical institution: 1. private clinic; 2. private hospital

C. retail pharmacy

D. shopping mall and supermarket pharmacy

2.9. Did he/she take any medicine during the acute illness, including medicines taken during hospitalization? **［ ］**

A. yes B. no (If the answer is “no”, skip to question 3.1)

2.10. Which medicines were taken during this illness?

|  | name of the medicine | Dosage form (1.tablet; 2. injection; 3. capsule; 4. granule; 5. oral liquid preparation; 6. external use; 7. other) | Who prescribed or recommended the use of the medicines? (1. doctor; 2. neighbor; 3. other household member; 4. patients themselves) | Where did he/she get the medicines? (1. public medical institution; 2. private medical institution; 3. retail pharmacy; 4. shopping mall and supermarket pharmacy) |
| --- | --- | --- | --- | --- |
| medicine 1 |  |  |  |  |
| medicine 2 |  |  |  |  |
| medicine 3 |  |  |  |  |
| medicine 4 |  |  |  |  |
| medicine 5 |  |  |  |  |
| medicine 6 |  |  |  |  |
| medicine 7 |  |  |  |  |
| medicine 8 |  |  |  |  |
| medicine 9 |  |  |  |  |
| medicine 10 |  |  |  |  |

2.11. Did he/she take all prescribed or recommended medicines? **［ ］**

A. yes B. no

2.12. If the answer is “no”, what is the reason? 【Multiple choice】 **［ ］**

A. prices of medicines are too high

B. the shortage of medicines

C. it is far from the purchase point of the medicines, and it is not convenient to buy

D. side effects of medicines

E. the way of taking medicines and taste etc. are not acceptable

F. other reasons, such as excessive amount of prescription medicines

2.13. How much did you pay for the medicines used to treat this acute illness?

yuan

2.14. Was this cost covered by medical insurance? **［ ］**

A. yes (2.14.1 reimbursed or paid yuan)

B. no

1. **Use of medicines in patients with chronic illness**

3.1. Has anyone in the household ever been told by a doctor or other health care provider that they have a chronic illness? (A chronic illness is an illness that will not go away or takes a long time to go away, even when treated. It mainly includes heart disease, hypertension, diabetes, apoplexy, chronic gastritis, etc.) **［ ］**

A. yes B. no (If the answer is “no”, skip to question 4.1)

3.2. How many persons in the household have a chronic illness?

3.3. What is the age of the oldest person with a chronic illness?

3.4. What is the gender of the oldest person with a chronic illness? **［ ］**

A. male B. female

3.5. Which illness does he/she have? 【Multiple choice】 **［ ］**

1. hypertension; 2. heart disease; 3. diabetes; 4. asthma; 5. arthritis; 6. high cholesterol;

7. chronic gastritis; 8. epilepsy; 9. apoplexy; 10. pulmonary tuberculosis; 11. liver disease;

12. depression; 13. cancer; 14. other

3.6. Has this person ever visited a health care facilities or providers for the chronic disease? **［ ］**

A. yes B. no

3.7. Has this person been told by a doctor or other health care provider that he/she should be taking medicines to treat this illness? **［ ］**

A. yes B. no

3.8. Which medicines has he/she been told to take for this chronic disease and for any other condition?

|  | name of the medicine | illness (refer to question 3.5) | Who prescribed or recommended the use of the medicines? (1. doctor; 2. neighbor; 3. other household member; 4. patients themselves) | the number of days of supply usually purchased/obtained at a time | how much was spent for the medicine last month | was this cost covered by medical insurance? (1.yes; 2.no) | personal payment  (refers to the cost of personal actual payment after deducting the cost of health insurance reimbursable) |
| --- | --- | --- | --- | --- | --- | --- | --- |
| medicine 1 |  |  |  |  |  |  |  |
| medicine 2 |  |  |  |  |  |  |  |
| medicine 3 |  |  |  |  |  |  |  |
| medicine 4 |  |  |  |  |  |  |  |
| medicine 5 |  |  |  |  |  |  |  |
| medicine 6 |  |  |  |  |  |  |  |
| medicine 7 |  |  |  |  |  |  |  |
| medicine 8 |  |  |  |  |  |  |  |
| medicine 9 |  |  |  |  |  |  |  |
| medicine 10 |  |  |  |  |  |  |  |

3.9. Some people cannot take all medicines as directed. Does he/she usually take all medicines as recommended? **［ ］**

A. yes B. no

3.10. I am going to give you possible reasons why the sick person may not always take medicines as recommended. Can you tell me whether these were reasons why he/she does not take medicines? 【Multiple choice】 **［ ］**

A. prices of medicines are too high

B. the shortage of medicines

C. It is far from the purchase point of the medicines, and it is not convenient to buy

D. Side effects of medicines

E. The way of taking medicines and taste etc. are not acceptable

F. Other reasons, such as excessive amount of prescription medicines

1. **Use of medicines in the household**

4.1 Do you have any medicines available at home today? **［ ］**

A. yes (4.1.1 there are  kinds of medicines)

B. no

4.2. Can I please see all of them?

|  | name of the medicine | medicine manufacturer (1. domestic medicines; 2. imported medicines) | where did you get the medicines? (1. public medical institution; 2. private medical institution; 3. retail pharmacy; 4. shopping mall and supermarket pharmacy) | why the household  has this medicine (1. the need to take sick 2. daily reserves 3. other reasons) | is the medicine’s label intact? (1. yes; 2. no) | is the medicine’s primary package intact? (1. yes; 2. no) |
| --- | --- | --- | --- | --- | --- | --- |
| medicine 1 |  |  |  |  |  |  |
| medicine 2 |  |  |  |  |  |  |
| medicine 3 |  |  |  |  |  |  |
| medicine 4 |  |  |  |  |  |  |
| medicine 5 |  |  |  |  |  |  |
| medicine 6 |  |  |  |  |  |  |
| medicine 7 |  |  |  |  |  |  |
| medicine 8 |  |  |  |  |  |  |
| medicine 9 |  |  |  |  |  |  |
| medicine 10 |  |  |  |  |  |  |

1. **Cognition of the quality of health care and medicine**

**I will make the following points for you. There is no standard answer to these questions. You just need to say whether you agree with the following points.**

5.1. Accessibility of health care and medicine:

A. full agreement B. partial agreement

C. no agreement D. no knowledge

5.1.1. Is the position of a public medical institution very convenient? **［ ］**

5.1.2. Is there a reasonable time in public medical institutions? **［ ］**

5.1.3. Are the required medicines available in public medical institutions? **［ ］**

5.1.4. Are the required medicines available in retail pharmacy? **［ ］**

5.2. The affordability of medicine:

A. full agreement B. partial agreement

C. no agreement D. no knowledge

5.2.1. Is the price of medicine in the public medical institution cheaper than the price of the private medical institution? **［ ］**

5.2.2. Is the price of medicine in the public medical institution cheaper than the price of the retail pharmacy? **［ ］**

5.2.3. Can your household fully afford all the cost of the medicine that the household needs?**［ ］**

5.2.4. Will you increase the use of medicines after you buy health insurance? **［ ］**

5.2.5. Have you ever received free medicines from the public medical institution? **［ ］**

A. yes B. no

5.2.6. Have your household borrowed or sold property to pay for medicine charges? **［ ］**

A. yes B. no

5.3. The quality of health care and medicine:

A. satisfied B. general

C. dissatisfied D. no knowledge

5.3.1 Are you satisfied with the health care provided by local public health institutions? **［ ］**

5.3.2 Are you satisfied with the health care provided by local the private medical institution? **［ ］**

5.3.3 Do you think the quality of branded medicines is better than the quality of non-branded medicines? **［ ］**

A. yes B. no C. no knowledge

5.3.4 Do you think the quality of imported medicines is better than the quality of domestic medicines? **［ ］**

A. yes B. no C. no knowledge

1. **The economic situation in household**

6.1. Can you please tell me how many rooms there are in your household? **［ ］**

A. two rooms, one halls and below B. two rooms, two halls

C. three rooms, two hall D. four rooms , two halls and more

6.2. In the past year, what was the average monthly income of your household?

6.3. Do you have the following items in your household?

| TV | refrigerator | air conditioner | washing machine | computer | car | house property | total score |
| --- | --- | --- | --- | --- | --- | --- | --- |
|  |  |  |  |  |  |  |  |

6.4. In the last week, how much did your household spend on food? yuan

6.5. In the last 4 weeks, what was your total household expenditure? yuan

6.6. In the last 4 weeks, how much did your household spend on health and medicines?

Health yuan Medicines yuan

6.7. What is your gender?

A. male B. female

6.8. What is your age?

A.44 years old and below B.45-59 years old

C.60-74 years old D.75 years old and above

6.9. What is the highest level of school education you passed? **［ ］**

A. junior middle school and below B. high school and higher vocational education

C. college and undergraduate D. graduate students and above
